# Biodentine^TM^ Full Pulpotomy in Mature Permanent Teeth with Irreversible Pulpitis and Apical Periodontitis

**DOI:** 10.3390/healthcare9060720

**Published:** 2021-06-12

**Authors:** Xuan Vinh Tran, Lan Thi Quynh Ngo, Tchilalo Boukpessi

**Affiliations:** 1 Faculty of Odonto-Stomatology, University of Medicine and Pharmacy at Ho Chi Minh City (UMP), 217 Hong Bang, 11 Ward, 5 District, Ho Chi Minh City 70000, Vietnam; ngothiquynhlan@ump.edu.vn; 2 UR 2496 Laboratory of Orofacial Pathologies, Imaging and Biotherapies, School of Dentistry, Université de Paris, 1 rue Maurice Arnoux, 92120 Montrouge, France; 3 AP-HP Department of Dental Medicine, Charles Foix Hospital, 94200 Ivry sur Seine, France

**Keywords:** vital pulp therapy, tricalcium-silicate-based cement, full pulpotomy, irreversible pulpitis

## Abstract

Vital pulp therapy, including direct pulp capping and partial and full pulpotomy, is primarily indicated for immature or mature permanent teeth with reversible pulpitis. Mature permanent teeth with irreversible pulpitis are frequently treated with root canal therapy. This report presents two cases of full pulpotomy using Biodentine^TM^ in mature permanent teeth with irreversible pulpitis and acute apical periodontitis. The periapical radiograph illustrated a deep carious lesion extended to the pulp with apical radiolucency lesion or widened periodontal ligament space. Full pulpotomy with a tricalcium silicate-based cement was chosen as the definitive treatment. After decayed tissue excavation under a rubber dam, the exposed pulp tissue was amputated to the level of the canal orifice with a new sterile bur. Biodentine^TM^ was applied as the pulp capping agent after hemostasis was obtained and for temporary restoration. The clinical signs disappeared quickly after the treatment. After one month, the coronal part of the temporary restoration was removed, and a composite resin was placed over the capping agent as a final restoration. At two-year follow-ups, the teeth were asymptomatic. Radiographs showed healing of the periapical lesion and periodontal ligament. Biodentine^TM^ full pulpotomy of mature permanent teeth with irreversible pulpitis and apical periodontitis can be an alternative option to root canal therapy.

## 1. Introduction

The maintenance of the vitality of the dental pulp is one of the crucial targets of modern dentistry, based on the concept of minimally invasive dentistry. The dental pulp presents capacity for repair, depending on the intensity of damage and the pulp inflammation. Two regenerative mechanisms, categorized as tertiary reactionary and reparative dentinogenesis, are involved in maintaining the vitality of the dentin–pulp complex. In the case of a carious lesion with relatively slow progression, the molecules that initially reach the pulpal tissue are able to induce dentin regeneration [1]. The dentin can be regenerated as odontoblasts, which are located on the periphery of the mature pulp and solely responsible for dentin synthesis. These can up-regulate their secretory activity and produce a thick layer of reactionary dentin. This layer shows many similarities to the primary and secondary physiological dentins and contributes to the protection of the pulp tissue. Reactionary dentin synthesis is promoted by small amounts of pro-inflammatory cytokines and/or biologically active molecules responsible for the induction of embryonic odontoblast differentiation, such as TGF or BMP [2]. Reactionary dentin formation is inhibited by intense inflammation [3]. In response to a severe injury, such as a rapidly progressing carious lesion, the primary odontoblasts die beneath the lesion [4]. It is hypothesized that bacterial toxins, components released from the demineralized dentin, or local generation of high levels of proinflammatory mediators, cause this event. Subsequently, however, if conditions become conducive (e.g., if the carious infection is controlled or arrested), stem/progenitor cells within the pulp are signaled to target the site of the injury and to differentiate into odontoblast-like cells. These cells deposit a tertiary reparative dentin matrix, reportedly at a similar rate to that of primary dentinogenesis, and this clinically results in dentin bridge formation [5].

Vital pulp therapy (VPT), which includes direct pulp capping and partial or full pulpotomy of exposed pulp in carious teeth, has been generally accepted as a minimally invasive approach [6,7]. Until recently, the indication of VPT had been reversible pulpitis in immature or mature teeth without periapical pathologies. Most cases of closed-apex permanent teeth with irreversible pulpitis are frequently treated with nonsurgical root canal therapy (RCT). If periapical signs and symptoms are added, RCT is the treatment of choice [8,9]. In this procedure, there is loss of dental hard tissue and subsequent weakening of the treated tooth, making it more susceptible to fracture [10]. Furthermore, several studies have highlighted that the actual failure rate of standard root canal treatments performed in general practice is significantly higher than expected [11,12,13]. Moreover, these treatments are lengthy and costly, and are often subject to retreatment [14]. Therefore, less invasive alternative strategies could be used to treat pulpitis, even when irreversible. Many biological and clinical studies have shown that the pulp of mature teeth, which is exposed due to carious lesions, is able to be regenerated, and that VPT should not be limited only to young or asymptomatic teeth. Therefore, a more conservative approach of VPT has been proposed for teeth with irreversible pulpitis. A favorable outcome of this approach depends on two factors: the healing ability of the remaining vital pulp and the biocompatibility of the pulp-capping agents used [15,16,17,18].

Mineral trioxide aggregate (MTA) is the optimal choice when VPT needs to be carried out in closed apex teeth [19,20,21]. The ability of MTA to induce reparative dentinogenesis has been well demonstrated in animal studies in which direct pulp-capping was performed in mechanically exposed pulps [22,23]; compared with calcium hydroxide, MTA induces dentin formation at a greater rate and with a superior structural quality [24]. However, many complaints have been made regarding the difficulty of handling and mixing MTA, the long setting time, and tooth discoloration over time [25]. Several new calcium silicate-based materials have been developed [26,27], aiming to address the disadvantages of MTA [28]. Biodentine^TM^ (Septodont, Saint Maur des Fosses, France) is among these materials, and is claimed to be able to be used as a dentin replacement material, in addition to having endodontic indications similar to those of MTA. Biodentine^TM^ is resin-free and mainly composed of pure tricalcium silicate, which is able to set in wet conditions [29]. Biodentine^TM^ has been shown to induce odontoblastic differentiation of dental pulp stem cells, and produce more uniform and thicker dentin bridge formations, with less inflammatory response and less necrosis of pulp tissue than calcium hydroxide [23,30].

The role of vital pulp therapy in the management of periodontal disease presenting in adult permanent teeth with irreversible pulpitis is controversial. The two cases below present the outcome of full pulpotomy, using Biodentine^TM^, of permanent teeth with irreversible pulpitis and periapical lesion/widened periodontal ligament space.

## 2. Case Presentation

**Figure 1:**
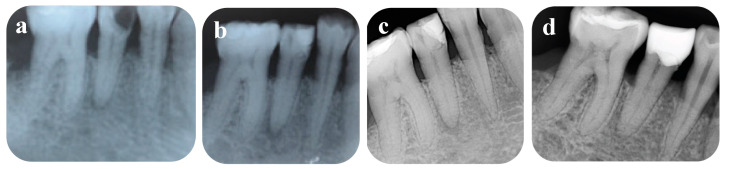
Periapical radiographs: (**a**) preoperative; (**b**) after treatment; (**c**) 6 months postoperative; (**d**) 12 months postoperative.

Case 1:

A 40-year-old female patient expressed her chief complaint as her spontaneous and lingering pain, pain on chewing in tooth number 45, starting one month previously. Clinical examination recorded that the affected tooth had a large carious lesion and sensitivity to percussion. The periapical radiograph illustrated a deep carious lesion involving the pulp and an apical translucency lesion (Figure 1). Based on the clinical and radiographic examinations, the diagnosis was established as symptomatic irreversible pulpitis. The patient consented to the full pulpotomy treatment plan.

**Figure 2:**
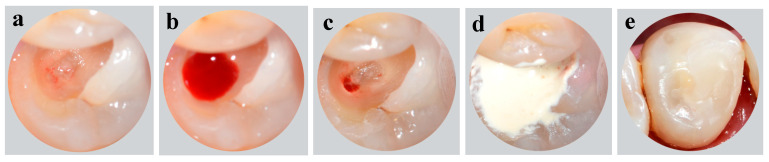
Intraoral photographs: (**a**–**c**) access opening for pulpotomy procedure; (**d**) placement of Biodentine^TM^; (**e**) composite resin restoration.

The tooth was anaesthetized with 2% Lidocaine Hydrochloride and Epinephrine 1:100,000 (Septodont, Saint-Maur-des-Fosses Cedex, France) before the placement of a rubber dam for isolation. The operating site was disinfected with gauze soaked in 5% sodium hypochlorite (NaOCl). Decayed tissues were removed using a sterilized high-speed round bur under water coolant. Then, the exposed pulp tissue was amputated by a sterilized high-speed round bur to the level of the canal orifice. The bleeding was arrested after about two minutes by gently pressing a sterile cotton pellet soaked in 2.5% sodium hypochlorite (NaOCl) into the chamber. The cavity was then filled with freshly prepared Biodentine^TM^ (Septodont, Saint-Maur-des-Fosses Cedex, France) using an amalgam carrier, and gently pressed with a condenser (Figure 2). The patient was asked to return after one month unless progressive pain occurred.

At the next appointment, the patient reported that mild pain occurred on the first post-treatment day, but the pain was soon alleviated. Moreover, vertical percussion inflicted no pain. The superficial layer of Biodentine^TM^ was removed, leaving a layer of approximately 3 mm. The tooth was finally restored with composite resin (3M ESPE, St Paul, MN, USA). Clinical and radiographic evaluation was completed at 6 months and 1 year postoperatively (Figure 1). The patient had no complaint about the tooth, and negative responses to cold and electric pulp tests, and periapical radiographs showed no periapical lesion after 1 year. At a 6-month follow-up examination, gaps were radiographically observed at the tooth–resin composite interface, so the old filling was replaced by an overlay composite restoration.

**Figure 3:**
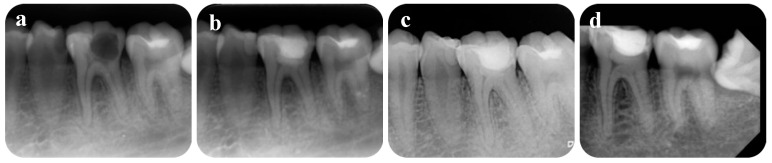
Periapical radiographs: (**a**) preoperative; (**b**) after treatment; (**c**) 6 months postoperative; (**d**) 24 months postoperative.

Case 2:

A 25-year-old female patient presented with a main complaint of severe spontaneous and lingering pain in tooth number 36, occurring several times over the previous two weeks. Pain was provoked by chewing or cold drinks. Clinical examination recorded caries extending to the pulp tissue, and the tooth was also sensitive to vertical and horizontal percussion. Periapical radiograph demonstrated widened periodontal ligament space at the mesial root (Figure 3). The tooth was diagnosed with irreversible pulpitis.

After receiving the informed consent from the patient, the same procedure as above was applied. The coronal pulp was removed to the level of the canal orifices. Bleeding was confirmed from all root orifices. After hemostasis was obtained, the pulp chamber was filled with Biodentine^TM^ as a capping agent and temporary restorative material.

The patient reported mild pain on the operation day, but the pain was reduced from the following day. One month later, the patient did not feel discomfort upon chewing, although vertical percussion caused a slight pain. The superficial layer of Biodentine^TM^ was removed, then the tooth was permanently restored with composite resin. After 6 months, there was no sensitivity to percussion and the periodontal ligament space improved. A 24-month examination indicated the periodontal ligament space had returned to the normal state, the tooth had no symptoms, and showed negative responses to cold and electric pulp tests.

## 3. Discussion

Until recently, the remedy for irreversible pulpitis has been endodontic treatment. Non-surgical endodontic treatment is considered to be an invasive and non-biological treatment because it removes the entire inflamed, infected, and healthy pulp, thus losing its reparative/regenerative potential, proprioceptive properties, and innervation [31]. Therefore, a more conservative approach with VPT has been proposed for teeth with irreversible pulpitis [15,16,17,18]. 

The successful outcome of both cases provides additional clinical evidence of the effectiveness of full pulpotomy in teeth with clinical signs and symptoms of irreversible pulpitis with apical periodontitis. Taha et al. (2017) reported that the success rate of MTA pulpotomy in mature permanent teeth presenting carious pulp exposures was 100% at one-year follow-up, and 92.7% after three years [20]. In another prospective study on Biodentine^TM^ involving full pulpotomy in mature permanent teeth with irreversible pulpitis, the authors found a high clinical success rate after one year of close to 100%, and a radiographic success of up to 93.8% [32]. Cushley et al. (2019) evaluated the clinical success rate of full pulpotomy in permanent teeth with signs and symptoms of irreversible pulpitis by a systematic review. They found a success rate of full pulpotomy of 97.4% clinically and 95.4% radiographically at 12-month follow-up [33]. However, VPT for mature permanent teeth with irreversible pulpitis and periapical lesion remains controversial.

In the current case report, the adult patients had spontaneous pain, lingering pain, and percussion sensitivity, which have long been clinical predictors of the irreversible stage of the pulp [34]. Furthermore, radiographically, these teeth presented a carious deep lesion and apical lesion or widened periodontal ligament space. In both cases, clinical signs and symptoms improved one month after Biodentine^TM^ full pulpotomy. We also recorded complete radiographic healing. In the first case, the apical radiolucency was improved after 6 months and completely healed after 12 months. The periodontal ligament space in the second case was in a normal state after 6 months.

The pulp tissue can remain vital, even in teeth with the presence of periapical radiolucency; this vital pulp tissue has the potential to recover in the presence of an adequate material [35]. Periapical inflammatory responses are related to the diffusion of bacterial products into the periapical tissue, causing a complex interaction of inflammatory mediators, cytokines, and neuropeptides [35]. Studies have shown that apical periodontitis can be associated with irreversible pulpitis. The finding of apical periodontitis in radiographic images does not necessarily mean that the pulp is necrotic. The inflamed vital dental pulp causes an immunological response, which could lead to local changes in peri-apical connective tissues [17,36,37]. Hence, clinical signs and symptoms of the patient do not reflect the actual extent of inflammation in the pulp tissue. In addition, the healing of teeth with irreversible pulpitis and a peri-apical lesion following vital pulp therapy has been demonstrated in few studies [11,18,32]. A widened periodontal ligament via an infectious pathway was reported in teeth with pulpitis, pulpo-periapical lesions, or even vital pulps with minimal hyperemic involvement [38,39]. However, the management of periodontal ligament widening in the teeth with irreversible pulpitis has rarely been mentioned in previous studies.

Accurate clinical diagnosis is significant in VPT, but it has been shown that clinical examination gives only a temporary diagnosis that may be incorrect [40,41]. The control of bleeding after removal of the infected pulp tissue has been suggested as an additional diagnostic indicator for the evaluation of the degree of inflammation and the healing potential of the remaining pulp tissue [15,42]. The ability to control bleeding within 5–10 min suggests the presence of mild to moderate inflamed pulp, which can heal in a conducive environment [18]. In both cases, bleeding occurred within 2 min, thus indicating VPT.

In our case report, Biodentine^TM^ was used as a pulp capping agent. Our previous in vivo studies demonstrated that Biodentine^TM^ provides an optimal environment for pulp healing, inducing the formation of a homogeneous dentin bridge at the injury site when applied directly to mechanically exposed rat pulps. In fact, the dentin matrix-associated growth factors can signal mesenchymal stem cells in the pulp to differentiate into odontoblast-like cells and produce a mineralized barrier in continuity with the primary dentin protecting the underlying vital pulp tissue [23,43].

A histological study found that the pulp tissue a few millimeters from the necrotic pulp with bacterial colonization is usually free from inflammation and bacteria [41]. The radicular pulp is rarely inflamed. Therefore, as soon as the infected and inflamed tissue is removed and an appropriate capping agent is applied, a favorable environment for pulp wound healing is created. In addition to its good sealing properties, Biodentine™, like other cements in the tricalcium silicate family, is able to control pro-inflammatory factor secretion and decrease inflammatory cell recruitment [44].

Long-term failure after vital pulp therapy and endodontic treatment is mainly attributed to micro-leakage at the coronal tooth–restoration interface. Massler et al. (1978) demonstrated that the most important cause of long-term failure in vital pulp therapy is the presence of leakage during the healing process [45]. Biodentine^TM^ presented good sealing ability, resisting micro-leakage [46], and its bond strength when bonded to resin composite was improved at a maturation time of 2 weeks [47]. Biodentine^TM^ has been shown to improve setting time, handling, and mechanical properties, compared with MTA [48]. This cement can be used successfully in dental clinics as a restorative material for up to 6 months, and as a dentin substitute under a composite for posterior restoration [49].

Success assessment of VPT is based on clinical and radiographic follow-up. The tooth should be asymptomatic. The tooth with full pulpotomy is expected to be unresponsive to sensibility testing. However, it should be positive to testing in the case of pulp capping or partial pulpotomy. A negative response does not indicate pulp necrosis. Success is defined as the absence of symptoms and maintenance of pulp vitality after a least 1 year [50].

## 4. Conclusions

Based on the perspective of bioactive material and pulp biology, full pulpotomy in mature permanent teeth with irreversible pulpitis, and apical periodontitis or widened periodontal ligament space might be considered as an alternative treatment to root canal treatment. Longer-term study is needed to confirm the future benefits of this treatment option.
